# Mitral shifting in arythmia complicated with bilateral renal infarction

**DOI:** 10.11604/pamj.2021.38.182.27389

**Published:** 2021-02-17

**Authors:** Chtioui Mamoun, Benameur Brahim

**Affiliations:** 1 Département de Cardiologie Interventionnelle, Centre Médico-Chirurgical, Agadir, Maroc,; 2 Service des Urgences, Centre Médico-Chirurgical, Agadir, Maroc

**Keywords:** Heart attack, valve disease, kidneys

## Image in medicine

We report the case of a 30-year-old woman with no individual's history, suffering from chest pain recently discovered; admitted to our department for dyspnea of rest. The clinical examination finds signs of global heart failure, with auscultatory signs of mitral stenosis in atrial fibrillation. The electrocardiogram records an arrhythmia tachycardia by atrial fibrillation (A). Trans-thoracic echocardiography demonstrated tight fibrous mitral stenosis, atrial dilatation and discrete left ventricular (LV) dysfunction, the right heart is without abnormality (B). As soon as she was put on treatment by digitalo-duiretic, and anticoagulated, the patient presented a brutal of intense bilateral abdominal pain radiating towards the flanks. An injected abdominopelvic computed tomography (CT) showed a bilateral acute occlusion of both renal arteries on the right (C). The patient had endovascular treatment with thromboaspiration. The interest of this observation is the rarity of this thromboembolic complication of mitral valve disease.

**Figure 1:**
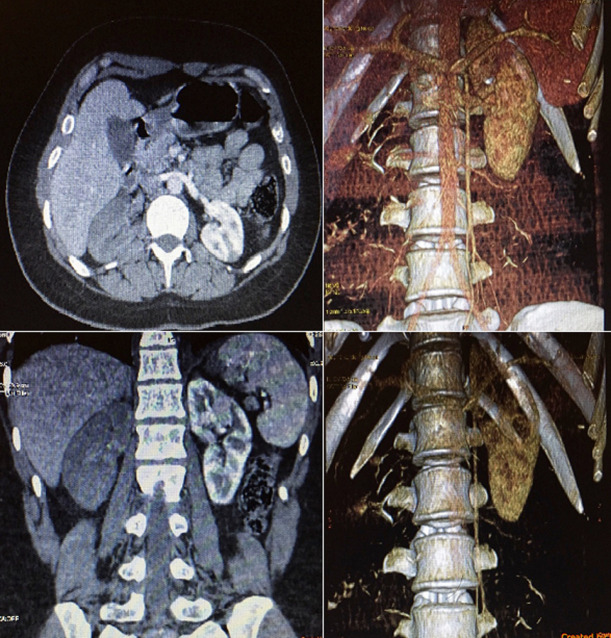
CT pelvic abdominal injected showing total renal infarction right and partial left confirmed at 3D volume-rendering-image

